# The Approach to the COVID-19 Pandemic in Georgia—A Health Policy Analysis

**DOI:** 10.3389/ijph.2022.1604410

**Published:** 2022-05-03

**Authors:** Ilia Nadareishvili, Ana Zhulina, Aleksandre Tskitishvili, Gvantsa Togonidze, David E. Bloom, Karsten Lunze

**Affiliations:** ^1^ David Tvildiani Medical University, Tbilisi, Georgia; ^2^ Independent Researcher, Tbilisi, Georgia; ^3^ Department of Global Health and Population, School of Public Health, Harvard University, Boston, MA, United States; ^4^ School of Medicine, Boston University, Boston, MA, United States; ^5^ Boston Medical Center, Boston, MA, United States

**Keywords:** COVID-19, health policy, policy analysis, politics, Georgia

## Abstract

**Objectives:** This study aimed to analyze key COVID-19 pandemic-related policies and national strategic responses in light of Georgia’s political, socioeconomic and cultural backgrounds.

**Methods:** We applied a policy triangle framework for policy analysis, performed document and media content analysis, and described pandemic trends statistically.

**Results:** Early introduction of stringent restrictive measures largely prevented a first wave in March–May 2020. This was communicated as a success story, prompting a public success perception. With unpopular restrictions lifted and hesitancy to embrace evidence-informed policymaking ahead of nationwide parliamentary elections, SARS-CoV-2 infection spread rapidly and was met with an insufficiently coordinated effort. Facing health system capacity saturation an almost complete lockdown was re-introduced in late 2020. Factors as delayed immunization campaign, insufficient coordination and, again, little evidence-informed policymaking eventually led to another devastating COVID-19 wave in summer of 2021.

**Conclusion:** Georgia’s pandemic health policy response was adversely impacted by a volatile political environment. National pandemic preparedness and response might benefit from an independent body with appointment procedures and operations shielded from political influences to effectively inform and communicate evidence-based pandemic policy.

## Introduction

The COVID-19 pandemic, with almost have a billion reported cases as of April 2022, has taken over 6 million lives, has had unprecedented health effects with high morbidity and health complications such as long COVID syndrome, and adversely impacted socioeconomic development of populations around the world. Coordination and management of response and mitigation efforts (on international or local levels) have revealed deep systematic challenges. New strains and outbreaks have continued to emerge, and some of them are highly virulent. Despite similarly unprecedented vaccination efforts, it is yet unclear when the pandemic will transition to an endemic. It seems obvious though that tens of millions more will contract the disease and millions more will die through future waves of the disease.

Documentation and analysis of health policy responses to COVID-19 on national and international levels is an active research processes across the globe. Comparative analyses, despite challenges associated with performance of such comparative studies can help optimize COVID-19 response strategies and prepare for future health crises [1–6].

Both the COVID-19 response and the outcomes of pandemic waves differed substantially among countries and territories, with weaknesses that manifested in many health systems soon after the beginning of the pandemic [7]. Georgia is an Eastern European country of 3.7 million inhabitants located in the geopolitically volatile South Caucasus, bordering Russia, Azerbaijan, Armenia and Turkey. Albeit at times among the countries worst affected by the pandemic globally (by per capita confirmed cases and deaths), Georgia’s COVID-19 experience and the country’s related policies have received limited attention yet. This study aimed to analyze key COVID-19 pandemic-related policies and national strategic responses in light of Georgia’s political, socioeconomic and cultural backgrounds.

## Methods

We conducted a health policy analysis using the policy triangle framework, which is grounded in a political economy perspective that considers the content of policy, actors, context and processes. We analyzed all aspects through document and content analysis [8–12].

To better understand the COVID-19 response in Georgia, we performed a qualitative content analysis using an inductive coding and analysis approach [13]. We searched for relevant documents (policy documents available online, such as government reports, programs, action plans and legal documents, as well as from non-governmental organization reports) published in the period from March 2020 to July 2021. We conducted the search from April 2021 to July 2021. We also searched official websites (including but not limited to www.gov.ge, www.moh.ge, www.stopcov.ge, www.ncdc.ge) and media content related to COVID-19 in Georgia. We selected the following media forms (restricted to material available online): social media text and video content, other video content (e.g., videos from TV resources, such as interviews or briefings), and online podcast/radio audio content. In our search for media content, we used a purposive sampling approach, focusing on social media pages of official government bodies (the prime ministers, Government of Georgia, the MoIDPLHSA, etc.) and several established media web pages and social media pages. Additionally, we focused on content produced by or on behalf of several national high-level policy makers and COVID-19 response leaders (e.g., the prime ministers, the minister of health, the deputy ministers of health, leadership of the NCDC, other key response persons). Considering the variety of content and topics, we formed several primary categories (with such labels as: “COVID-19 Regulations,” “Restrictive Measures,” “Economic Anti-Crisis Plan,” “Responsibility Attribution” etc.) and focused on content falling in the categories of our primary interest. We also took other relevant resources into consideration. We excluded quotations without a video/audio proof to avoid “fake news”, unless the resources were posted on official governmental web or social media pages (e.g., www.stopcov.ge). We also excluded duplicate resources reporting identical text, quotes or information across various media resources. The final media content dataset (after discarding duplicating and irrelevant sources) included 352 text and 112 video resources. We then performed an exploratory content analysis of the retrieved data. The data set included the following information: “Source of Information” (e.g., NCDC, Media News Report), Date, Internet Link, “Speaker” (who’s quote does the text contain; in case of institutional statement—name of institution), main content text or message, and finally the category code(s). First, two researchers performed a pilot “blind” coding and reviewed the code list. We then manually coded the content (video/audio content was first “live coded” [14], followed by partial transcription (e.g., reducing the content, avoiding impractical data volumes, such as daily statistical updates or content irrelevant to policy analysis) and text coding). The categories and codes we used are given in the Supplementary Material S1. Some pertinent qualitative data was translated to English. Two of the authors were not fluent in Georgian, which limited their quantitative data analysis input to analysis of documents, reports, publications that were originally available in English and those additional text sections which were partially translated from Georgian to English by the Georgian team members (all of them fluent in English).

Additionally, we compiled epidemiological data from publicly available resources (www.ncdc.ge, ourworldindata.org) in this study’s databases. The NCDC data sets included the following data: daily confirmed SARS-CoV-2 positive cases and COVID-19-related deaths, cumulative cases and deaths and the daily number of tests conducted, etc. We complemented the NCDC data sets by calculating rates of positive tests per numbers of tests conducted and adding restrictive measures’ stringency index and COVID-19 vaccination related data (obtained from www.ourworldindata.org). The final quantitative data set is available as Supplementary Material S1. We used descriptive statistics and graphical visualization to assess pandemic trends in Georgia and describe these trends in the context of the response policies. Using the data published by NCDC we estimated differences in mortality by patient age and sex categories.

### Ethical Issues/Statement

The study did not involve any primary human data or human subjects and therefore didn’t require ethics approval.

## Results

### Context

A former Soviet Union state, Georgia declared independence in 1991, and joined the United Nations in 1992. Before the pandemic, Georgia’s three international airports had direct connections to dozens of international destinations, including major transit hubs. Still, most international travellers arrive and depart through land borders. According to the World Bank, in 2019 (before the pandemic) Georgia’s nominal GDP was about 17.5$ billion. Its GDP per capita (4,439$ in 2019) is just marginally above the threshold for upper-middle-income economy status [15]. Georgia’s developing economy is highly susceptible to pandemic disruptions given its reliance on international visitors, tourism and personal remittances, as well as foreign direct investment, trade and services [16, 17]. Over a million citizens (28% of the total population) live in households registered as socioeconomically vulnerable, half of which receive financial social support from the government [18].

The country’s decentralized healthcare system, mostly privatized during the post-Soviet public health reform period of 2007–2010, serves both urban and rural populations with a high burden of risk factors for severe forms and complications of COVID-19 (e.g., obesity, cardiovascular diseases, diabetes and other non-communicable conditions). Despite recent increases in healthcare spending, Georgia’s total annual per capita spending on health has remained at the equivalent of just over 300 USD since about 2015. While almost 5 hospital beds per 1,000 population are available, the primary care sector is underdeveloped and lacks gatekeeping capacity, as people in Georgia rarely visit primary care physicians [19–21]. With 30,000 practicing physicians nationwide, including over 500 ICU specialists, Georgia has one of the highest physician rates per capita globally at 8 per 1,000 population. However, Georgia has a chronic shortage of nurses [19]. Insufficient medical education, including little availability of Continuing Medical Education and Continuing Professional Development, is another concern [22, 23].

Decades of collaboration with the World Health Organization (WHO) and the US Centers for Disease Control and Prevention (CDC) have significantly increased the country’s epidemiologic preparedness. This included the enhancement of communicable disease tracing and human and laboratory resources, further expanded through the foundation of the Richard G. Lugar Center for Public Health Research to support pandemic preparedness and response. Most other laboratory capacities are private.

From 2013, Georgia’s political system gradually shifted from a presidential to a parliamentary republic, with the transformation finalized in 2018. The prime minister and all 11 ministers were from one political party, the Georgian Dream party. Representatives of this party have formed the government of Georgia for many years. From 2016 to 2020, Georgian Dream held the constitutional majority in the parliament, with only two other political forces represented. Georgia has seen accentuated political polarization since around 2012 and lately a nearly continuous wave of protests since June 2019. The country faced the COVID-19 pandemic at a time of preparation for general parliamentary elections and amid political turmoil in the country and the region (e.g., the 2020 Nagorno-Karabakh war) [17]. The 2020 parliamentary elections, held during the pandemic, resulted in 9 political forces securing seats in the parliament. Due to extreme political polarization, it was not until April 2021 (following an agreement mediated by the EU and United States, which among others called on sides to work together to address the COVID-19 crisis and related challenges) that opposition parties have joined the parliament during the COVID-19 pandemic [24]. The political situation remained fragile though, as the agreement did not hold, continuing the deep political crisis in the country.

### Actors

The Government of Georgia, led by a Prime Minister, performed the key role in the pandemic response of making policy decisions for the culturally, socioeconomically and ethnically diverse people of Georgia throughout the crisis. Multisectoral actors in the government response to COVID-19 included the Ministry of Internally Displaced Persons, Labour, Health and Social Affairs (MoIDPLHSA) of Georgia, its subordinate National Center of Disease Control and Public Health (NCDC) and other ministries, i.e., of Internal Affairs, Economy and Finances. The pandemic policymaking process has been highly centralized at the national government level; regional, city and municipal governments had limited involvement in policymaking, but played an important role in the execution of the pandemic response. NCDC was the key research disease surveillance institutions, which conducted several epidemiologic studies (e.g., seroprevalence surveys), monitored circulating strains, etc. Research [25], official communications and educational interventions during the COVID-19 response were supported and guided by information campaigns from international bodies, such as the WHO, the CDC, United Nations Children’s Fund (UNICEF) and other governments and international organizations. Yet few local analytic and modelling studies were conducted in the country, and policy making heavily relied to expert opinions and foreign experience.

The main acting COVID-19 decision-making body in Georgia was the Interagency Coordination Council led by the Prime Minister of Georgia [26]. The Council brought together all cabinet ministers, including such key actors as representatives from the MoIDPLHSA, the Ministry of Internal Affairs, the Ministry of Economy, other government actors and two public health officials from the NCDC. The Council also regularly invited relevant key experts, e.g., intensive care specialists, infectious disease and public health experts and other public figures. Georgia’s opposition had no decision-making power (the ruling party enjoyed a two-thirds majority, completing the government cabinet while opposition parties boycotted parliament work for months) in the pandemic response until joining the national Parliament in May 2021 when it gained representation in parliamentary committees.

The Interagency Coordination Council’s rules and policies differed across regions in Georgia, most prominently through geographically varied entry-exit restrictions (e.g., temporary closure of roads in and out of the largest cities in April and May 2020, local quarantine zones in some hotspot municipalities) [27] and authorization of in-person classes in schools in rural areas and smaller towns. In May 2020, the Parliament of Georgia made the controversial decision to transfer decision-making power on such restrictive measures as “curfews” (“temporary restriction of outdoor and public space movement for anything but emergencies [still requiring short-term movement permits] in specific hours of a day”) to the executive branch, thus circumventing the legal requirement to declare a state of emergency in advance of implementing curfew measures [28].

### Content and Processes

#### Pandemic Trends in Georgia

**FIGURE 1:**
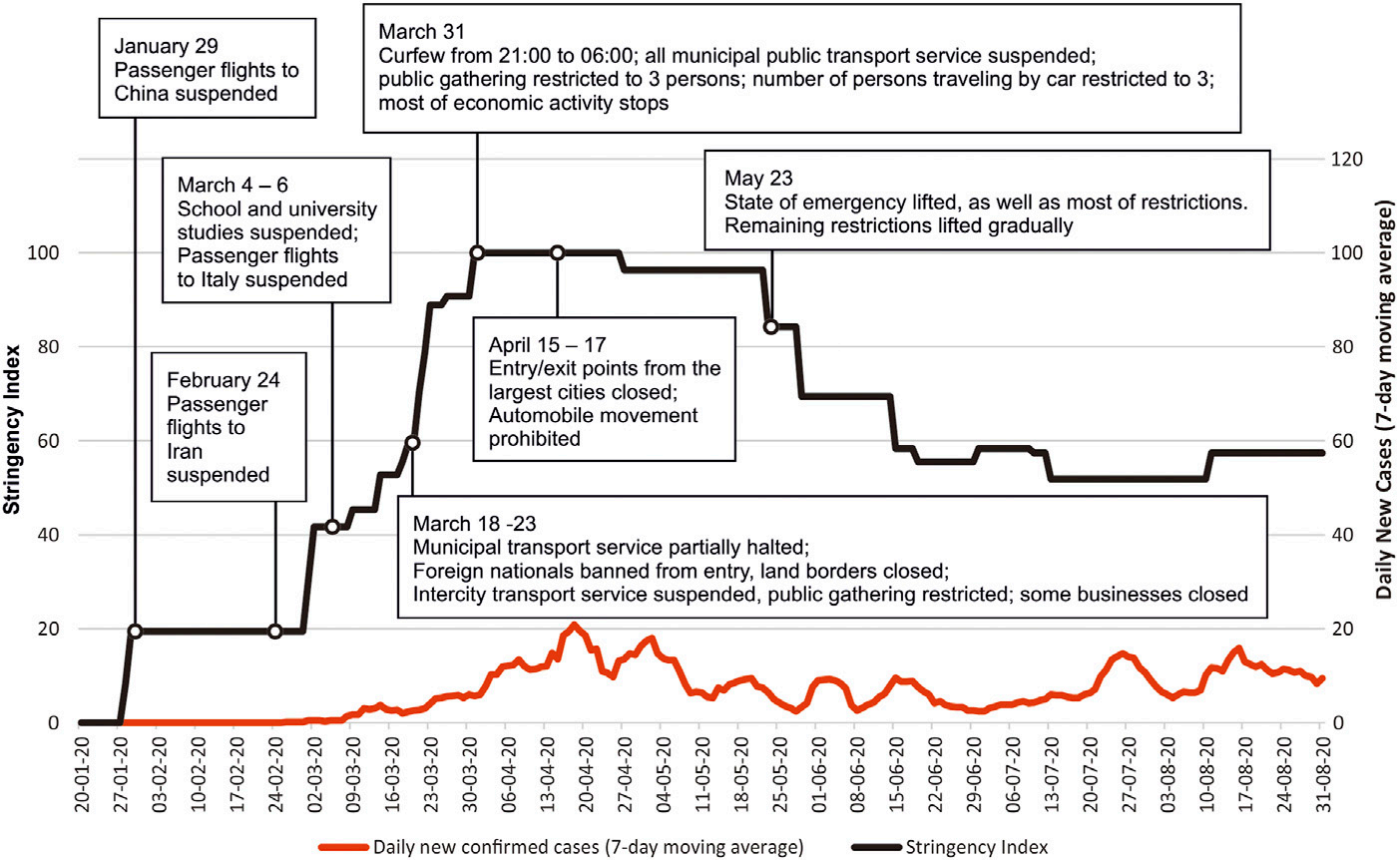
Daily confirmed cases 7-day moving average (February 2020—31 August 2020) and the “Stringency Index” (the stringency index is a composite measure based on nine pandemic response indicators e.g. school closure and travel bans, rescaled to a value from 0 to 100) with some of the key points influencing pandemic dynamics (Georgia, 2021).

Having recorded its first COVID-19 case on 26 February 2020 and initially facing very limited testing and clinical management capacities, the Georgian government promptly mandated restrictive measures, culminating in a 2-month strict lockdown and state of emergency starting 21 March 2020. A “curfew” (restriction of any outdoor movement from 21 p.m. through 06 a.m.) was enforced for most of April and May 2021 (see Figure 1). By mid-April 2020, the roads out of the four largest cities had been closed, movement of most of modes of private and public transport (cars) were prohibited and daily movements reduced by about 80% [27, 28]. The NCDC’s leadership appealed to the public to avoid Easter-related gatherings, with which most of the population complied, expressing a high level of trust in their leadership [29, 30]. At the same time, entrance in cemeteries was prohibited. With 721 confirmed cases and 12 deaths nationwide by the end of May 2020, Georgia managed to avoid a significant first wave, albeit at the substantial economic cost of hundreds of thousands of job losses and a severe recession (as described below under the Economic Impact sub-heading). This initial epidemiological success prompted initial perceptions of victory over the pandemic by the political leadership, which were communicated to the Georgian public as a success story and reported by some international media as such [27, 31, 32].

In response, a public health leader later warned against the “[…] illusion that the country defeated COVID infection–no, we did not. The victory over COVID is still far away and one single country can’t do that.” [33] (NCDC director general, 21 July 2020)

At the onset of the pandemic, the NCDC implemented intensive efforts on contact tracing and quarantine until capacity was exceeded. A network of “fever clinics” was formed for rapid assessment, isolation and testing of persons presenting a fever. Until the end of 2020, testing was targeted to symptomatic persons, healthcare personnel and other frontline workers.

**FIGURE 2:**
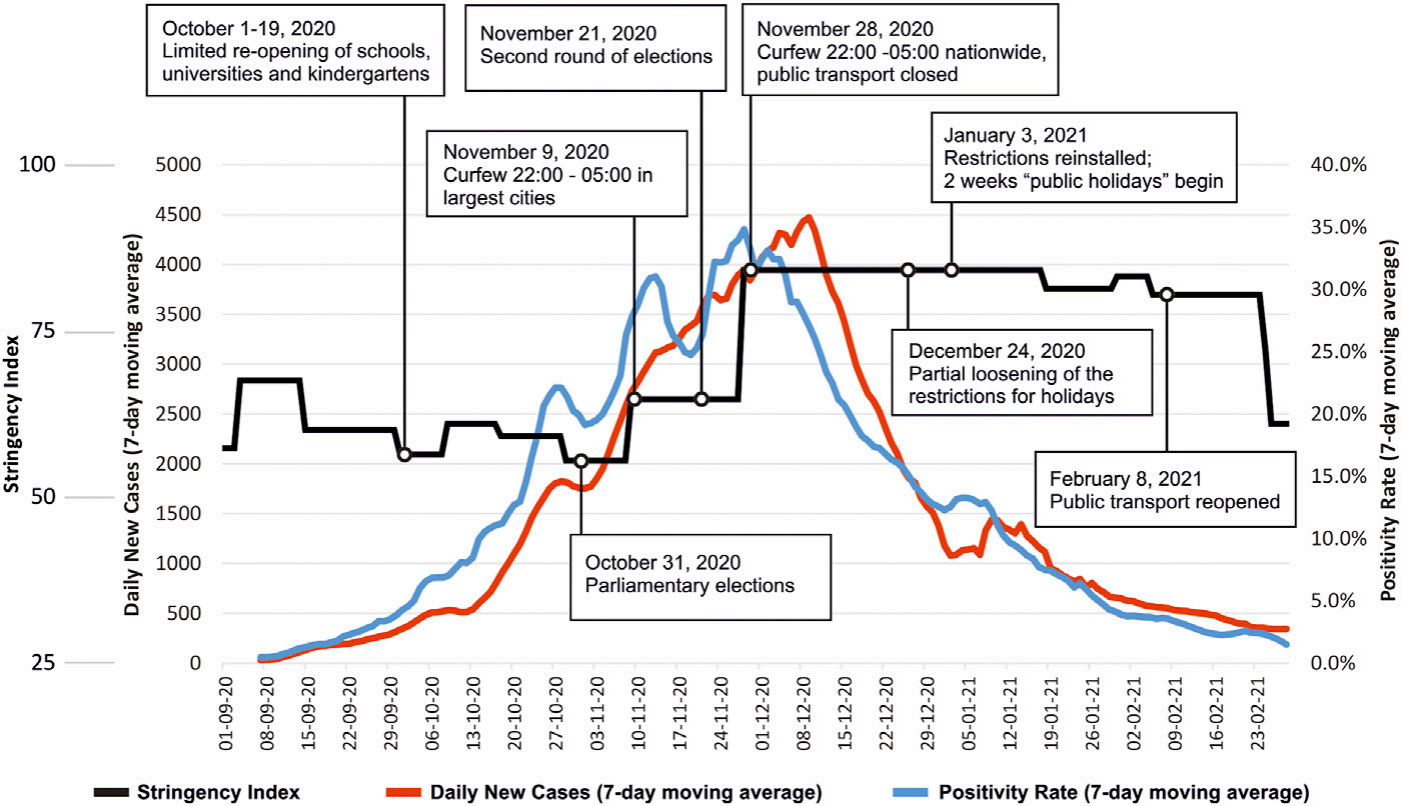
Daily confirmed cases 7-day moving average, percent positives (the percentage of all COVID-19 tests performed that returned positive results) 7-day moving average and the “Stringency Index” (the stringency index is a composite measure based on nine pandemic response indicators e.g., school closure and travel bans, rescaled to a value from 0 to 100) (1 September 2020—28 February 2021) with some of the key points influencing pandemic dynamics (Georgia, 2021).

In the meantime, the national government developed and mandated hygiene protocols and guidelines for implementation in hotels, child care facilities, secondary and higher educational institutions, shopping malls, markets and restaurants [34–40]. Case numbers remained low until the end of September 2020 due to limited cross-border movement and in spite of inconsistent enforcement of hygiene protocols and guidelines [28]. With restrictions mostly lifted over the period from June to September 2020, cases began to increase later that year. High tourist activity, low compliance with even milder restrictions and inconsistent enforcement resulted in a major outbreak, leading to over 250,000 people testing positive during the “second wave” of October 2020–January 2021 (see Figure 2). Retrospectively, the health minister regretted the summertime easing of restrictions: “Perhaps it was also my mistake as I was not demanding firmly enough to categorically keep the restrictions that had been instituted in spring” (minister of health, 24 November 2020) [41].

#### The Role of Political Processes

As the country prepared for parliamentary elections scheduled for 31 October 2020, which created additional mobility, the government was hesitant to reinstitute unpopular largescale restrictive measures. “[…] The political process comes first, which we won’t and can’t stop. Despite some so-called experts’ calls, these processes can’t be touched and we must all understand well that free, democratic elections, with a face-mask, keeping distance, this is a priority, with which Georgia should show the whole world that it is possible to continue normal life even in such a situation … ” (then Prime Minister, 7 October 2020) [42]. Certain observers expressed concern that the legal basis for the elections did not allow for adequate pandemic restrictions, and some government officials admitted that the pre-election period adversely impacted pandemic containment efforts.

Infection control measures were in place at voting stations to ensure safety of the elections [43]. However, the pre-election period of rallies and other campaign activities, and accompanying policymaking that did not consistently implement evidence-based preventive measures may have exacerbated the epidemic. An independent study estimated that at least 100,000 excess cases and 1,250 excess deaths were attributable to the elections [44].

#### Further Developments

After two rounds of elections and with new cases rising daily, the government announced a second, 2-month period of restrictive measures (rather than referring to it as “lockdown”, terming it instead as “targeted restriction,” though the COVID-19 Government Response Tracker Stringency Index was above 80 in the period 28 November 2020–23 February 2021) beginning 28 November 2020. Right before announcing the second “lockdown” the government held several consultancy meetings with health care experts and met representatives of several economy sector stakeholders (tourism, retail, restaurants, etc.). Meanwhile, the 14-day-incidence of new cases per 100,000 population, initially not surpassing 20, exceeded 1400 by mid-December 2020 (the highest in Europe and Asia at that point), with over 5,000 newly confirmed cases nationwide on several days [45]. Healthcare services exceeded their capacities at that point. Those requiring hospitalization were sometimes transported to other cities where hospital beds were available. Consequently, the cumulative number of deaths increased rapidly, reaching 3,500 at the end of February 2021 (i.e., 1 year after the first case was reported), up from 19 in mid-September and 700 in mid-November 2020. Excess mortality was observed during this period [45].

By mid-October 2020, Georgia faced health workforce overload and burnout. Medical schools were instructed to urgently prepare senior medical students for field or phone center work. Of over 1,700 trained medical students, about half joined the workforce ahead of their projected graduation. By the end of June 2021, 24,345 health workers in Georgia had been diagnosed with COVID-19 (and 76 of them, predominantly those over 60 years old, died). Approximately 30% of them were physicians and 40% were nurses (about a third of all practicing physicians and over half of employed nurses) [45].

A restriction of outdoor movement in evening-night hours imposed in November 2020 spanned the period between the second and third waves (until 30 June 2021, though the specific “curfew” hours were gradually reduced). The third wave’s peak coincided with Easter 2021, for which a 2-week period of leave from work and a temporary closure of public transport in the cities was implemented.

To supplement the few state-owned facilities, the government subcontracted private clinics to establish a network of “COVID clinics”, i.e., hospitals admitting and managing patients with or suspected of having COVID-19. Facing high patient loads during the second wave, physicians began providing primary assessments via telephone. These “call-center clinics” contributed significantly to patient triage, identifying those needing confirmatory testing or hospitalization. Furthermore a network of “Clinical Hotels” was established to isolate and monitor patients with mild symptoms, primarily those whose living conditions didn’t allow self-isolation. Hospitals’ expenses and treatment costs while functioning as “COVID clinics” were financed from the MoIDPLHSA budget [46].

#### COVID-19 Immunization

Georgia started immunizing its population on 15 March 2021, initially prioritizing healthcare workers and persons of over 75 years of age, gradually followed by other high-risk groups and later rolling out vaccination to the rest of populations. The plan aimed to fully vaccinate 60% of the adult population of about 1.7 million people by the end of 2021, about half of which were estimated to be in high-risk groups). According to this plan, full vaccination required 1 or 2 doses, depending on the specific vaccine, although all the vaccines which became available in Georgia in 2021 required at least 2 doses for full vaccination status protecting against the then dominant delta variant [47, 48]. Survey data indicated high rates of COVID-19 vaccine hesitancy in the population [49, 50]. Despite public information efforts of NCDC, academia and other actors, the vaccination campaign was criticized in the media for its delay due to vaccine unavailability (as well as uncertainties on vaccine availability timing) and insufficient communication of benefits, risks and procedures as well as little efforts to address the widespread anti-vaccination activity and disinformation. Early in the campaign, a young nurse with no prior risk factors who had gone on television to encourage people to get vaccinated just before getting her own injection died immediately following her vaccination. This contributed to very few healthcare workers and other eligible groups scheduling their vaccination appointments and resulted in excess vaccine dose availability. As a response, the planned allocation strategy had to be dropped and vaccination eligibility was extended to other risk groups, and starting in May 2021 to all adults. A striking preference for certain manufacturer brands, with more than 100,000 signing up for vaccination in only 90 min once Pfizer vaccines became widely available on 24 July. Despite the gradually increasing daily number of vaccine shots, just over 140,000 people (about 5% of the adult population and under 4% of the total population, about a quarter of those over the age of 65) were fully vaccinated with two doses by 28 July 2021.

#### Economic Impact

The pandemic brought about a GDP reduction of 6.2% in 2020 [51], a substantial depreciation of the national currency (the exchange rate was 2.8 GEL/USD in early March 2020 and over 3.3 a year later), a rise of those under the poverty line (from 42% to 46.6%) and a massive increase in unemployment (at least 350,000 people qualified for Government assistance of about 60$ per month for 6 months due to temporary or permanent job loss in the first half of 2020) [27, 52, 53]. The Georgian government undertook several waves of multibillion GEL interventions, which included making direct payments to people who had lost their income source or become bankrupt, as well as subsidizing a large part of utility bills, supporting the tourism sector and other measures [54]. Georgia managed to attract over 3 billion USD in foreign financial support (mostly in the form of foreign debt, but also direct financial support from EU and other foreign partners) with government debt surpassing 60% of GDP for the first time ever, while fiscal deficits increased significantly [15–17, 55, 56]. Economy strongly rebounded in 2021, though the year also saw higher than expected inflation levels as “Repeated waves of new COVID-19 infections threaten Georgia’s recovery” [16, 52].

#### Government’s Messages, Public Compliance and Attitudes

During the pandemic’s first wave, the Government of Georgia focused on spreading “stay home” messages through television, social media and other channels, reassuring the public that the healthcare system was capable of coping with the COVID-19 burden. Media gave a lot of coverage to several key persons responsible for epidemiologic and clinical care (infectious disease and intensive care specialists, etc.).

The period of June-August of 2020 saw a significant decline in interest in COVID-19. During this period, messages regularly stressed that the situation was under control. Officials kept a positive image until the very peak of the second wave, after the elections had been conducted [27]. The narrative then shifted to attribute the increase in the case numbers to people’s behaviors, such as holding mass gatherings like weddings and entertainment events, not wearing face masks and failing to keep physically distant. Terms like “regulation”, “law”, “recommendation”, “request”, and “advice” were used interchangeably, possibly confusing people about mandatory rules in place. Fake news and disinformation supporting conspiracy theories further affected public trust and adherence to local and international pandemic measures [57, 58].

#### The Fourth Wave

By early August 2021, a large-scale COVID-19 wave was on the rise, with the number of reported active infections surpassing 50,000 for the first time ever and counting. In parallel to the gradual growth in COVID-19 cases, the government lifted almost all existing restrictions and on 24 June (based on Prime Minister’s public statement, the document yet to be ratified), pardoned unpaid pandemic-related financial fines. The key policy as of late July 2021 was to speed up the delayed vaccination process and call on people to follow the general infection control recommendations, with a few regulations still remaining in force. As the political turmoil continued, the country’s leadership aimed to avoid another, potentially unpopular lockdown or widespread restrictions, citing the availability of vaccines and potentially devastating economic consequences as a reason. Entering August with low vaccination coverage, minimal restrictions, an unending political crisis, Georgia’s weak health system is once again put to test as mobilization of hospital beds and human resources continues.

**FIGURE 3:**
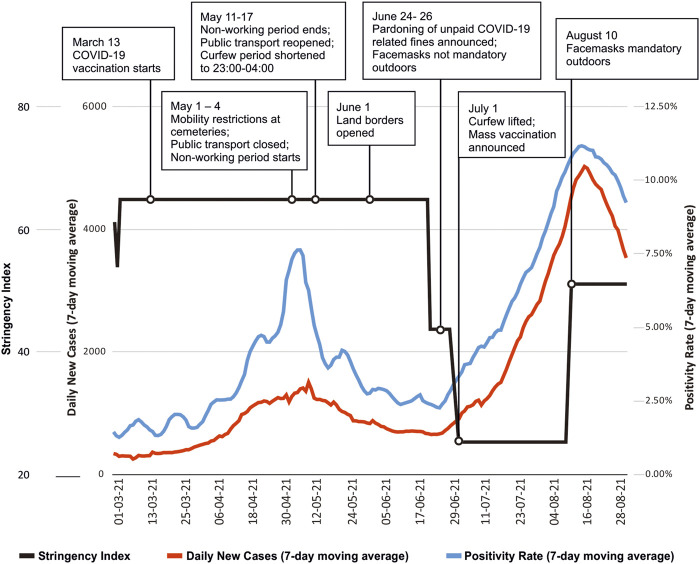
Daily confirmed cases 7-day moving average, percent positives (the percentage of all COVID-19 tests performed that returned positive results) 7-day moving average and the “Stringency Index” (1 March 2021—31 August 2021) with some of the key points influencing pandemic dynamics (Georgia, 2021).

As of 27 July 2021, the NCDC confirmed over 404,000 COVID-19 positive cases and more than 5,700 COVID-19-related deaths. The number of positive cases represents approximately 11% of Georgia’s total population. Persons aged ≥70 years had a 15-fold (95% CI: 14.5–16.2) higher mortality risk compared with those <70 years. Males were at increased risk of death compared to females. The 7-day moving average positivity rate in Georgia exceeded 5% by early October 2020 and rose to over 30% in November, gradually reducing to under 5% in December 2020 and January 2021 (following the introduction of the second round of “lockdown” scale restrictive measures from November 28, 2020, with the stringency index of over 80%). Despite significantly higher number of daily tests in 2021 compared to 2020, daily positivity rates still rose to over 10% by August 2021 (see Figure 3 and the Supplementary Material S1).

## Discussion

This health policy analysis assessed key policies of COVID-19 pandemic response in Georgia, in a complex context of local and regional political crisis and unique health system. . Our findings suggest that in spite of significant weaknesses, systematic problems and the pandemic’s toll, the Georgian health system, generally avoided a healthcare system collapse, resulting in relatively low mortality in the first pandemic wave (largely due to timely mobility restrictions). Considering that decisions in the first half of 2020 were taken in the context of uncertainty and low preparedness, the measures taken ahead of the second wave improved human and material healthcare resources, e.g., for testing and treatment capacity [30]. Similar to Georgia, the Czech Republic and Slovakia were among the countries that avoided a significant first wave but were eventually affected by substantial numbers of COVID-19 cases [59–61]. Previously perceived as inefficient, the country’s low hospital bed occupancy and the high number of available ICU beds became an asset for COVID-19 management in Georgia.

However, various factors, including general parliamentary elections and reluctance to re-impose unpopular restrictions, resulted in severe second and third waves of the pandemic. The decision to hold elections during the pandemic was not unique to Georgia, as about half of all countries in the world held elections under various infection control measures since March 2020 [62]. Policy making based on, or justified by, expert opinions could have been better informed by modeling studies to simulate different local pandemic scenarios. Modeling studies can thus inform adapted, targeted interventions to prevent further viral spread or respond to pandemic exacerbations. Several studies explored experts’ role in policy making, and it has been suggested that “the extent to which public policy responses to COVID-19 were based on health, political, or economic imperatives, and the extent to which politicians or unelected experts were to the fore in framing political messaging, varied considerably across Europe and had a major bearing on the nature and timing of decisions” [1, 63, 64]. It was noted early into the pandemic that the actual or perceived political dependence of scientific advisory groups which many countries established was a concern. While knowledge transfer from researchers to policy making bodies increased trust to the decisions, many experts where perceived as biased and too close to policy makers that appointed them, raising questions about transparency, independence, objectivity and rigour of the experts’ advice [65]. On the other hand, it has been known that health research capacities in Georgia are limited [66]. Insufficient coherence and consistence of the Georgian government’s communication around pandemic strategies limited public trust and adversely impacted compliance with restrictions [67–70]. Like many other countries, Georgia has struggled to timely obtain vaccines, and mounting a vaccination campaign also proved challenging for various reasons [71]. In preparation for future pandemics, a reimagining of global health governance may be necessary to ensure timely and adequate provision of vaccines, and to combat vaccine scepticism.

### Limitations

In this study, we did not analyse specific policies but rather provided an overview analysis of complex sets of pandemic related policies applied in Georgia, the uniqueness of the pandemic and its complexities. We did not conduct interviews with stakeholders nor did we collect other primary data, and we relied on publicly available document and media sources for content analysis. The COVID-19 pandemic has been characterized by rapidly evolving and changing situations and published data of varying quality. We recognize that not all printed or broadcast data is available online, as well as other limitations associated with media content analysis. Not all co-authors were fluent in Georgian; their contribution to quantitative data analysis relied on documents and reports available in English and the limited amount of translated qualitative data.

### Conclusion

Effective pandemic responses require evidence-informed policymaking to ensure public trust and adherence with pandemic measures, as well as balanced and consistent communication approaches and accountability. Pandemic health policy, like any policy, certainly always exists in a political context. To avoid adverse effects from volatile political processes like those seen in Georgia during the COVID-19 pandemic, countries might benefit from independent advisory bodies to inform and effectively communicate evidence-based pandemic policy. Providing binding guidance for national pandemic preparedness and response, such independent advisory bodies could foster knowledge sharing and equip governments with the best available data. Efforts should be made to ensure apolitical appointment of this body’s members, as well as insulation of its activities from political influences. This could support effective and targeted evidence-based pandemic policymaking and planning.
